# Emotional responses and perceived stressors of frontline medical staffs in case of COVID-19 treatment centers and obstetrics emergency in Ethiopia

**DOI:** 10.1186/s12888-021-03311-1

**Published:** 2021-06-15

**Authors:** Mebratu Abraha Kebede, Dereje Bayissa Demissie, Dessalegn Kenay Guddu, Michael Temane Haile, Zebenay Workneh Bitew, Mahteme Bekele Muleta

**Affiliations:** 1 ICCMH, Research Assistant officer, Research Directorate office, Saint Paul’s Hospital Millennium Medical College, Addis Ababa, Ethiopia; 2 Maternity and Reproductive Health, Director of Nursing education department, Saint Paul’s Hospital Millennium Medical College, Addis Ababa, Ethiopia; 3 Emergency medicine and Critical Care, Saint Paul’s Hospital Millennium Medical College, Addis Ababa, Ethiopia; 4 Nursing education department, Saint Paul’s Hospital Millennium Medical College, Addis Ababa, Ethiopia; 5 Saint Paul’s Hospital Millennium Medical College, Addis Ababa, Ethiopia

**Keywords:** Emotional responses, Perceived stressors, Front line medical staffs, Addis Ababa COVID-19 treatment centers

## Abstract

**Background:**

the rapid spread of COVID-19, its lethality in severe cases and the absence of specific medicine poses a huge threat to human life and health, as well as huge impact on the mental health. Facing this critical situation, health care workers on the front line who are directly involved in the diagnosis, treatment, and care of patients with COVID-19 are at risk of developing psychological distress and other mental health symptoms including emotional disturbance.

**Objective:**

the aim of this study will be to assess the current state of emotional responses and perceived stressors of frontline medical staffs in case of Addis Ababa COVID-19 Treatment Centers and obstetrics emergency and abortion care, Ethiopia 2020.

**Methods:**

Hospital based comparative cross-section study design was conducted by using self-administered questionnaire survey from June 1st to 30th of 2020 among 133 and 266 frontline medical staffs from obstetric emergency and abortion care clinic and COVID-19 treatment centers respectively. The data were collected after getting written consent from each participant and it entered into the computer using Epi-data version 7, then exported to SPSS version 20 for further analysis. Descriptive analysis was done using frequencies & percent. All independent determinants with *P*-value < 0.05 were used to identify important predictors of emotional responses and perceived stressors.

**Result:**

A total of 399 frontline medical staffs were included in the study. The mean age of the respondents of those who were working in obstetrics emergency and abortion care clinic was 27.47 (SD, 3.46) years and it was 28.12 (SD, 4.09) years for the other groups. This study revealed that, 72.9 and 5.6% of the study participant from obstetrics emergency and abortion clinic and COVID-19 treatment centers had a positive emotional response, respectively. Factors such as having a low level of motivational factors (AOR 2.78, 95% CI (1.13, 6.84)), being a nurse (AOR 10.53, 95% CI (1.31, 85.26)) and working at triage (AOR 8.61, 95% CI (1.15, 64.81))) had statistically significant association with negative emotional response.

**Conclusion:**

The current study revealed that a high proportion of front line a negative emotional responses had negative emotional response. Further, almost all of the medical staffs working in COVID-19 treatment centers and at obstetrics emergency and abortion care unit had perceived the outbreak related stressors. So, providing comprehensive psychological support is warranted for health care providers working in such kinds of department or units.

## Introduction

Corona Virus Disease 2019 (COVID-19) is a disease caused by a novel corona virus (2019-nCoV) which was primarily identified in Wuhan, China on December, 2019 [1]. It is highly infectious during the incubation period and can be transmitted from person to person through respiratory droplets, contact and aerosols [2]. To date (October 16th of 2020), more than 39,596,856.00 cases of COVID-19 are found globally, causing more than 1107, 374.00 confirmed deaths around the globe [3]. Due to the rapid spread of COVID-19, strong contagion, lethality in severe cases, and no specific medicine, it poses a huge threat to human life and health, as well as huge impact on the mental health of the general public like emotional disturbance [4].

Although infectious diseases elicit a wide range of emotional responses, not everyone experiences the same degree of emotional impact [5]. However, health care providers who are directly involved in the diagnosis and treatment as well as care of patients with COVID-19 are at risk of developing psychological distress and other mental health symptoms [2]. The ever-increasing number of suspected and confirmed cases, overwhelming workload as well as feelings of being inadequately supported, depletion of personal protection equipment and lack of specific drugs may all contribute to the mental burden like emotional disturbance of these health care workers. Previous studies have reported adverse psychological reactions to the 2003 SARS outbreak among health care workers [5–7].

Those health care workers feared contagion and infection of their close family experiencing high levels psychological distresses which could have long-term psychological implications [8]. Unrecognized and asymptomatic patients transferred to their family or close contacts can be alarming and causing potentially distressing and emotional turmoil for front line health providers [6, 8]. In general mental health problems are inevitable among front line health care providers [9–13]. So, providing psychosocial support, timely psychological assistance and training in coping strategies to those frontline HCWs has its own contribution in regulating emotional response and perceived stressors [14, 15].

However, there are no published data that shows the emotions and perceived stressors of front line medical staffs in Ethiopia. To address this gap, the current study was aimed to assess the level of emotional response and perceived stressor among frontline Medical Staffs’ from COVID-19 treatment centers and obstetrics emergency/abortion acre units in Addis Ababa city, Ethiopia.

## Methods

### Study area and period

The study was conducted from June 1st to 30th of 2020 among 133 and 266 frontline medical staffs from obstetric emergency and abortion care clinic and COVID-19 treatment centers of Eka Kotebe General Hospital, St. Paul’s Hospital Millennium Medical College (SPHMMC) and St. Peter specialized Hospital, Addis Ababa, Ethiopia. SPHMMC, a teaching Hospital located in the capital city, Addis Ababa. The current St. Peter ‘s hospital was established in 1963G.C as a TB treatment center in the nation. At the time of Haile Selassie, I, with the charity and goodwill of Knojit Anbenet, wife of Ras Abebe, the residence of the War Minister was given to Ministry of Health to serve as TB sanatorium. Eka Kotebe General Hospital in Addis Ababa has five-floor building that provides services of mental health with 150 beds and general medical services with 200 beds has been built with a total cost of 160 million Birr. A nine-floor building for administrative work and Mental Health Institute has also been inaugurated on the same day. Eka Kotebe General Hospital is under the auspices of the St Amanuel Mental Specialized Hospital. A total of 354 health care workers were assigned for COVID-19 treatment centers and 237 of HCW were working at obstetrics emergency and abortion care units of the selected three hospitals.

### Study design, study population and inclusion criteria

Hospital based comparative cross-sectional study was conducted among all frontline Medical Staffs who were working in Addis Ababa COVID-19 treatment Centers and obstetrics emergency and abortion care departments at least 2 weeks during the study period at Eka Kotebe General Hospital, St. Paul’s and St. Peter Hospital in Ethiopia.

### Sample size determination, sampling technique, data collection and analysis

Sample size was determined by considering of two population proportion by allocating of 1:2 ratio of front line medical staffs working at obstetrics emergency and COVID-19 treatment centers respectively. A simple random sampling technique was employed in order to select a representative sample of frontline Medical Staffs from each institution. Structured self-administered written questionnaires was administered to collect the data on the independent variables and outcome variables.

The study tools are comprehensive questionnaire derived and modified from the one used by Khalid, Khalid, Qabajah, Barnard, and Qushmaq 2016 for the hospital staff during the MERS-CoVepidemic [5]. We termed it “COVID-19 staff questionnaire” after having a discussion with senior internists and public health expertise staff members of saint Paul Hospital. Emotional response was the extent that they experienced anxious, fear, sadness and anger in response to the outbreak of COVID-19 in in Addis Ababa COVID-19 treatment Center and obstetrics emergency and abortion care clinic on a 4-point scale, ranging from 0 (no such emotion) to 3 (the most intense feeling of the emotion). The questionnaire consisted of 15 questions that explored staff emotions during the COVID-19 outbreak. Finally, level of emotional responses was classified based on the participants score which 25% and above was considered as having a negative emotional response, whereas those who will score below 25% were considered as having a positive emotional response. With respect to perceived stressors, those who scored below 25% of the perceived stressor related question were considered as having no stressor and those who scored 25% and above were classified under having perceived stressor. The tool used to assess level of perceived stressor had also 4-point scale (0 = very minimal, 1 = slight; 2 = moderate; 3 = very much). Further social support status was measured based on Oslo 3-item social support scale and those who scored greater than or equal to 9 [16]. Pre-test was conducted among 5 % of the sample size which was included seven health care providers from obstetrics emergency and abortion care unit of Ras Desta hospital and thirteen from Millennium COVID-19 treatment centers. During the pretest appropriateness of wording, clarity of the questions including for language and respondent reaction to the questions and interviewer were assessed.

Data was collected for one month, checked for completeness and consistency of responses manually. After cleaning it was entered, in to EPI-Data version 3.5.4 then exported to SPSS versions 25 for analysis. Bivariable and multivariable logistic analyses were performed to determine the presences of statistically significant associations between the independent variables and emotional responses and perceived stressor. All variables having a *p*-value < 0.25 in the bivariable analysis were selected for the multivariable logistic regression to control for possible confounders. Those variables which showed significant association on bivariable analysis are adjusted to each other to identify independent determinants. The strength of the association was presented by odds ratio and 95% confidence interval. Variables with a p-value of < 0.05 on multivariable analysis were considered as statistically significant factors.

### Ethical considerations

Ethical approval and clearance was obtained from SPHMMC ethical review board and supportive letter was written to Eka Kotebe general hospital, SPHMMC, and St. Peter. In addition, consent of the respondents was obtained after giving information and thoroughly explaining the aim of the study to each respondent. The participants were told that participation is voluntary, could withdraw any time or refuse to answer to any question if they want to. The questionnaire was self-administrated questionnaire to maintain privacy. No information concerning the individual was passed to a third party.

## Result

### Socio-demographic characteristics of the respondents

A total of 133 and 266 frontline medical staffs from obstetrics emergency and abortion care clinic and COVID-19 treatment centers respectively were included in this study. The mean age of the respondents for those who were working in obstetrics emergency and abortion care clinic was 27.47 (SD, 3.46) years as well as it was 28.12 (SD, 4.09) years for the other groups. In the case of the respondents from obstetrics emergency and abortion care clinic, the majority 66 (49.6%) were in age range of 26–30 years and 94 (70.7%) were male also, 95 (71.4%) were single. Further, 59 (44.4%) of them were Resident/Intern in profession and 70 (52.6%) & 129 (97%) were working at delivery unit and saint Paul hospital millennium medical college (SPHMMC) respectively. In addition, 112 (84.2%) of them had no child, as well as 88 (66.2%) were not living with family at time of outbreak. Furthermore, 69 (51.9%) had emergency obstetric/ abortion care treatment and prevention related training and 68 (51.1%) had intermediate level of social support. (Table 1).

**Table 1:** Socio demographic, professional and social support related description among frontline medical staffs in case of obstetrics emergency and abortion care clinic, Addis Ababa, Ethiopia, 2020

Variables		Frequency	Percent (%)
Age	25 years and below	44	33.1
	26–30 years	66	49.6
	31 and above years	23	17.3
Sex	Male	94	70.7
	Female	39	29.3
Marital status	Married	38	28.6
	Single	95	71.4
Place of work	Triage	6	4.5
	Delivery unit	70	52.6
	Abortion clinic	2	1.5
	Obstetrics operation room	5	3.8
	Emergency OBY/GNY	50	37.6
Profession	Nurse	9	6.8
	Midwifery	42	31.6
	Physician (GP)	6	4.5
	Obstetric Specialist	10	7.5
	Gynecology specialist	7	5.3
	Resident/Intern	59	44.4
Clinical experience	2 years and below	80	60.2
	3–4 year	25	18.8
	5 year and above	28	21.1
Employer institution	YekaKotebe Hospital	2	1.5
	SPHMMC	129	97.0
	St. peter Specialize Hospital	2	1.5
Have a child	Yes	21	15.8
	No	112	84.2
Living with family at time of outbreak	Yes	45	33.8
	No	88	66.2
Have ever trained in emergency obstetric/ abortion care treatment and prevention	Yes	64	48.1
	No	69	51.9
Social support	Poor social support	53	39.8
	Intermediate social support	68	51.1
	Good social support	12	9.0

With respect to the study participants in the case of COVID-19 treatment centers, the majority which accounts 137 (51.5%) were in age range of 26–30 years and 155 (58.3%) were male and single with a frequency of 177 (66.5%). Further, 219 (82.3%) were Emergency medicine and Critical Care Specialist in profession and 143 (53.8%) as well as 170 (63.9%) were working at clinical care unit and saint Paul hospital millennium medical college (SPHMMC) respectively. In addition, 195 (73.3%) of them had no child and 148 (55.6%) were not living with family at time of outbreak. Also, 205 (77.1%) had no COVID-19 treatment and prevention related training and 155 (58.3%) had poor level of social support. (Table 2).

**Table 2:** Socio demographic, professional and social support related description among frontline medical staffs in case of COVID-19 treatment centers, Addis Ababa, Ethiopia, 2020

Variables		Frequency	Percent (%)
Age	25 and below years	75	28.2
	26–30 years	137	51.5
	31 years and above	54	20.3
Sex	Male	155	58.3
	Female	111	41.7
Marital status	Married	83	31.2
	Single	177	66.5
	Divorced/widowed	6	2.3
Place of work	Critical Care Unit	143	53.8
	Emergency Medicine	75	28.2
	Outpatient Family Medicine	35	13.2
	Ward	5	1.9
	Triage	8	3.0
Profession	Emergency medicine and Critical Care Specialist	219	82.3
	Physician (GP)	44	16.5
	Nurse	3	1.1
Clinical experience	2 years and below	89	33.5
	3–4 years	82	30.8
	5 years and above	95	35.7
Employer institution	YekaKotebe Hospital	46	17.3
	SPHMMC	170	63.9
	St. peter Specialize Hospital	46	17.3
	Volunteers	4	1.5
Have a child	Yes	71	26.7
	No	195	73.3
Living with family at time of outbreak	Yes	118	44.4
	No	148	55.6
Ever trained in COVID-19 treatment and prevention	Yes	205	77.1
	No	61	22.9
Social support	Poor social support	155	58.3
	Intermediate social support	111	41.7

### Level of emotional response among front line medical staffs

**Fig. 1:**
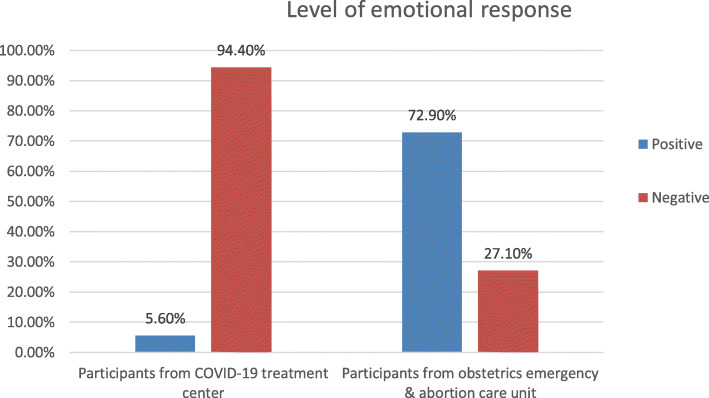
Level of emotional response among front line medical staffs working at obstetrics/abortion clinic and COVID-19 treatment centers, Addis Ababa, Ethiopia, 2020

This study revealed that, 72.9% (CI 66.2, 80.5) and 5.6% (CI 3, 8.3) of the study participant from obstetrics emergency and abortion clinic and COVID-19 treatment centers had positive emotional response respectively. (Fig. 1) The Cronbach’s alpha coefficient of emotional response categories of obstetrics emergency and abortion clinic and COVID-19 treatment centers are 0.704 and 0.743 respectively. The mean was 19.88 for medical staffs from obstetrics emergency and abortion care unit and it was 22.58 for those from COVID-19 treatment center.

### Level of perceived stressor among the study participants

**Fig. 2:**
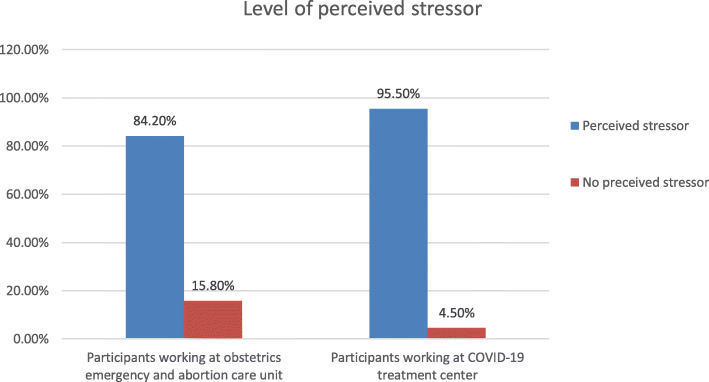
Level of perceived stressor status among front line medical staffs working at obstetrics/abortion clinic and COVID-19 treatment centers, Addis Ababa, Ethiopia, 2020

This study revealed that the level of perceived stressor was 84.2% (CI 77.4, 89.5) and 95.5% (CI 92.9, 97.7) among frontline medical staffs working at obstetrics emergency and abortion care unit and at COVID-19 treatment centers respectively. The Cronbach’s alpha result was 0.764 and 0.903 for perceived stressor of the study participants working at COVID-19 treatment centers and obstetrics emergency/abortion care units, respectively. The mean for health workers from obstetrics emergency and abortion care unit and COVID − 19 treatment center was 35.19 and 42.09 respectively. (Fig. 2).

### Factors associated with emotional response and perceived stressor among frontline medical staffs

For each explanatory variable, bivariate analysis was done and factors such as place of working unit, profession and motivational status among frontline medical staffs working at obstetrics emergency and abortion care unit and factors such as social support and motivational factors among those who are working at COVID-19 treatment centers were variables fulfilled the minimum requirement at *p*-value < 0.05 significance level for further multivariate logistic analysis of emotional response. Whereas emotional response was the only factor which fulfill the minimum requirement at p-value less than 0.05 during the bivariate analysis for further multivariate analysis with perceived stressor among health care providers working at obstetrics emergency and abortion care unit. But, there was no factor which had an association with perceived stressor during the bivariate analysis among those who are working at COVID-19 treatment centers.

During the multivariate analysis for emotional response; factors motivational factors, profession and place of working unit had statistically significant associated with low level of emotional response among health care providers working at obstetrics emergency and abortion care unit; whereas only social support status had statistically significant association with emotional response among those frontline healthcare providers working at COVID-19 treatment centers. By using multivariate logistic regression, who had low level of motivational factors (AOR 2.78, 95% CI (1.13, 6.84)), nurses (AOR 10.53, 95% CI (1.31, 85.26)) and who were working at triage (AOR 8.61, 95% CI (1.15, 64.81))) had statistically significant association with low level of emotional response among health care providers working at obstetrics emergency and abortion care units. Further, those who had low social support (AOR 8.02, 95% CI (1.01, 64.00)) had statistically significant association with negative emotional response during the multivariate logistic regression among front line medical staffs working at COVID-19 treatment centers. (Tables 3 and 4).

**Table 3:** Factors associated with emotional response among frontline medical staffs working at obstetrics emergency/abortion care unit and COVID-19 treatment centers, Addis Ababa, Ethiopia, 2020

Explanatory Variables	Emotional response		COR,95%(CI)	AOR,95%(CI)	*p*-value
	Positive response	Negative response			
Place of work (a^1^)					
Triage	2	4	7.09, (1.44, 43.96)*	8.61,(1.15, 64.81)**	0.037
Delivery unit	49	21	1.52, (0.66, 3.53)	1.50, (0.57, 3.95)	0.417
Abortion clinic	2	0	0.00,(0.00, 0.00)	0.00,(0.00, 0.00)	0.999
Obstetrics operation room	5	0	0.00,(0.00, 0.00)	0.00,(0.00, 0.00)	0.999
Emergency OB/GYN	39	11	1	1	
Profession(a^1^)					
OBY/GYN specialist	15	2	1	1	
Nurse	4	5	9.38,(1.30, 67.65)*	10.53, (1.31, 85.26)**	0.027
Midwifery	26	16	4.62, (0.93, 22.89)	3.68, (0.68, 20.09)	0.132
Intern/GP/Resident	52	13	1.88, (0.38, 9.25)	1.86, (0.35, 10.00)	0.470
Motivational level (a^1^)					
low level of motivation	12	121	2.13, (1.09, 4.69)*	2.78,(1.13, 6.84)**	0.026
high level of motivation	3	130	1	1	
Social support (b^2)^					
Poor/low social support	14	141	10.92,(1.41,84.34)*	8.02, (1.01, 64.00)**	0.049
Intermediate social support	1	110	1	1	
Motivational status (b^2^)					
low level of motivation	12	121	4.30, (1.18, 15.60)*	0.36, (0.10, 1.36)	0.133
high level of motivation	3	130	1	1	

* Significant association (p-value < 0.05 in bivariate) **-significant association (*p*-value< 0.05in multivariate analysis);

**Table 4:** Factors associated with emotional response among frontline medical staffs working at obstetrics emergency/abortion care unit and COVID-19 treatment centers, Addis Ababa, Ethiopia, 2020

Explanatory Variables	Emotional response		COR,95%(CI)	AOR,95%(CI)	*p*-value
	Positive response	Negative response			
Place of work (a^1^)					
Triage	2	4	7.09, (1.44, 43.96)*	8.61,(1.15, 64.81)**	0.037
Delivery unit	49	21	1.52, (0.66, 3.53)	1.50, (0.57, 3.95)	0.417
Abortion clinic	2	0	0.00,(0.00, 0.00)	0.00,(0.00, 0.00)	0.999
Obstetrics operation room	5	0	0.00,(0.00, 0.00)	0.00,(0.00, 0.00)	0.999
Emergency OB/GYN	39	11	1	1	
Profession(a^1^)					
OBY/GYN specialist	15	2	1	1	
Nurse	4	5	9.38,(1.30, 67.65)*	10.53, (1.31, 85.26)**	0.027
Midwifery	26	16	4.62, (0.93, 22.89)	3.68, (0.68, 20.09)	0.132
Intern/GP/Resident	52	13	1.88, (0.38, 9.25)	1.86, (0.35, 10.00)	0.470
Motivational level (a^1^)					
low level of motivation	12	121	2.13, (1.09, 4.69)*	2.78,(1.13, 6.84)**	0.026
high level of motivation	3	130	1	1	
Social support (b^2)^					
Poor/low social support	14	141	10.92,(1.41,84.34)*	8.02, (1.01, 64.00)**	0.049
Intermediate social support	1	110	1	1	
Motivational status (b^2^)					
low level of motivation	12	121	4.30, (1.18, 15.60)*	0.36, (0.10, 1.36)	0.133
high level of motivation	3	130	1	1	

* Significant association (p-value < 0.05 in bivariate) **-significant association (*p*-value< 0.05in multivariate analysis);

a^1^ – factors associated emotional response among HCP working at obstetrics emergency/abortion care unit.

b^2^- factors associated emotional response among front line medical staffs working at COVID-19 treatment centers.

a^1^ – factors associated emotional response among HCP working at obstetrics emergency/abortion care unit.

b^2^- factors associated emotional response among front line medical staffs working at COVID-19 treatment centers.

## Discussion

The current study revealed that 72.9% of frontline medical staffs from obstetrics emergency and abortion clinic and 5.6% of those who are from Addis Ababa covid-19 treatment centers had positive emotional response. This implies health care workers working at COVID-19 treatment has more likely to have negative emotional response as compared to those who are working at obstetrics emergency and abortion clinic. That might be due to fear of acquiring the infection. Health professionals working at COVID-19 treatment center has high levels of burnout and psychological symptoms during the COVID-19 emergency which could have its own contribution to experience of negative emotion [17].

In other word 94.4% of the current study participants in the case of COVID-19 treatment centers had negative emotional response toward the outbreak which is higher than the previous study findings which was done in Italia indicated that 37 and 22.9% of the study participants had moderate and severe level of emotional exhaustion respectively [18]. According to the other study which was conducted in the hospitals of the Istituto Auxologico Italiano, 35.7 and 31.9% had moderate and severe levels of emotional exhaustion that is markedly lower than the current finding [17].

High level of negative emotional response might have associated because of health professionals experiencing emotional contagion that perceive higher stress which over longing period can cause burnout. Emotional contagion and perceived stressor could increase the risk of burnout that could be expressed with negative emotion amongst health care providers [19].

In addition, the current study showed that 84.2 and 95.5% of frontline medical staffs working at obstetrics emergency and abortion care unit and at COVID-19 treatment centers respectively had perceived stressors. This indicated that, more or less, it was in line on health care providers working in both units. This is supported by the idea that globally, 90% of the HCP reported somewhat or substantially higher levels of stressors [20]. However, the current study result was higher than the previous study finding reports such as in Wuhan more than half means 59.0% of the HCP had moderate to severe levels of perceived stress [21] and in India which 17.4%, 78.9 and 3.7 [22], also 25.5, 22.9 and 37% [18] had low, moderate and high level of perceived stressors respectively. Furthermore, it was also higher than the previous result that has been determined 54.5% of nurses and midwives have been making their lives worse since the outbreak started, 62.4% had difficulties in dealing with the uncertain situation in the outbreak [23]. The variation might be due to the presence or absence of personal protective equipment’s as well as other advanced medical infrastructures. The availability of strict infection control guidelines and specialized equipment provided psychological benefit especially in reduction stressors [24]. Ongoing and old pressures from their personal life can affect the emotional expression of the HCP in their day to day job that their mood may change such as increased irritability and feel chronically exhausted that further persist as a stressor [25].

Regarding, the associated factors, the current study showed that those who were nurses were 10.53 times more likely to experience negative emotional response as compared to those OBY/GYN specialists. This is supported by the previous study findings which reported that being a nurse as predictor of emotional exhaustion [17, 24] and shows more affective symptoms [18, 26]. So, timely counseling services and support systems should be given to those groups would help to mitigate the massive impact of the pandemic emergency on their actual and future emotional wellbeing [18].

With respect to place of working unit, those who were working at triage were 8.61 times more likely to have negative emotional response than those working at OBY/GYN emergency unit. This might be due to being as the first individual to be exposed with the patient and those who are working at the OBY/GYN emergency unit contact the patient after evaluated by the HCP working at triages. In addition, the current study identified that those who had low level of motivational factors were 2.78 times more likely to have negative emotional response as compared to those who had high level of motivational factors. This is augmented by the previous report that the presence of different motivating factors has a great contribution in reduction psychological impact like emotional disturbance of COVID-19 pandemic [24].

Among those front line health care providers working at COVID-19 treatment centers who had low social support were 8.02 times more likely to have negative emotional response than those who had good social support. This might be because of unavailability of proper support from their nearby individuals. The study which was done China mentioned that receiving negative feedback from families and friends contributes in the development of negative emotional response [27]. Further, having better social support might serve as a buffer against the dysfunctional consequences of stress emanating from the workplace and established network of friends, family, superior, peers, and colleagues to seek emotional support when faced with job-related stress in the workplace [28] but it was in contrast with other finding that emotional response has no relation with presence or absence of family support [8].

## Conclusion

The current study revealed that high proportion of front line medical staffs working at COVID-19 treatment centers (94.4%) and at obstetrics emergency and abortion care unit (27.1%) had negative emotional response. Further, almost all of the medical staffs working at COVID-19 treatment centers (95.5%) and at obstetrics emergency and abortion care unit (84.2%) had perceived the outbreak related stressors. Those health care providers who had low level of motivational factors, nurses, working at triage and had low social support were more experienced negative emotional response. So, providing comprehensive psychological support is warranted for those HCP working in such kinds of department or units.

## Data Availability

Raw data were generated at Eka Kotebe General Hospital, SPHMMC and St. Petter Specialized Hospital. Derived data supporting the findings of this study are available from the corresponding author MA and co-author DB, ZW, DK, MT and MB on request. This is also to confirm you that there is hardcopy of ethical approval letter that we have got it from SPHMMC IRB committee after the research proposal had been reviewed and approved. The collected hardcopy questionnaires are available with the principal investigator DB, whereas the softcopy of SPSS data is currently available among some of the co-investigators such as MA, ZW, MT, DK and MB by keeping it in confidential.
